# Monitoring bovine dairy calf health and related risk factors in the first three months of rearing

**DOI:** 10.1186/s13028-023-00708-8

**Published:** 2023-10-12

**Authors:** Nina Dam Otten, Alice Puk Skarbye, Mogens Agerbo Krogh, Anne Marie Michelsen, Liza Rosenbaum Nielsen

**Affiliations:** 1 Department of Veterinary and Animal Sciences, Faculty of Health and Medical Sciences, University of Copenhagen, Grønnegårdsvej 8, DK-1870 Frederiksberg C, Denmark; 2 Department of Animal and Veterinary Sciences, Aarhus University, Blichers Allé 20, DK-8830 Tjele, Denmark

**Keywords:** Calf health, Dairy calves, Disease monitoring, Epidemiology

## Abstract

**Background:**

Rearing replacement heifers is pivotal for the dairy industry and is associated with high input costs for the preweaned calves, due to their higher susceptibility to diseases. Ensuring calf health and viability calls for systematic approaches in order to mitigate the costs induced by managing sick calves and to ensure animal welfare. The objective of this study was to develop a systematic and feasible health-monitoring tool for bovine dairy calves based on repeated clinical observations and diagnostic results of calves at three time points; the 1st (T0), the 3rd (T1) and the 12th (T3) week of age. The study included observations from 77 dairy heifer calves in nine Danish commercial dairy herds. Immunisation status was assessed by serum Brix% at T0. Clinical scoring included gastrointestinal disease (GD) and respiratory disease (RD). The average daily weight gain (ADWG) was estimated from heart-girth measurements. Pathogen detection from nasal swabs and faecal samples were analysed for 16 respiratory and enteric pathogens by means of high-throughput real time-PCR. All measures obtained in each herd were visualised in a panel to follow the health status of each calf over time.

**Results:**

The individual clinical observations combined with diagnostic information from immunisation and pathogen detection form each enrolled calf are presented in a herd dashboard illustrating the health status over the study period. This monitoring revealed failure of passive transfer (Brix% < 8.1) in 31% of the 77 enrolled calves, signs of severe GD peaked at T0 with 20% affected calves, while signs of severe RD peaked at T2 with 42% affected calves. ADWG over the first eight weeks was estimated to be 760 g (± 190 g). Pathogen profiles varied between herds.

**Conclusions:**

The large variation in both clinical disease and pathogen occurrence across herds emphasizes the need for herd specific monitoring. Combining the results of the present study from measures of immunisation, health and growth from individual calves in one visualisation panel allowed for the detection of patterns across age groups in the specific herds, showing promising potential for early detection and interventions that can lead to enhanced calf health and welfare.

## Background

Monitoring dairy calf health is crucial to safeguard the animal welfare and production potential, and to reduce antimicrobial use on-farm in the dairy sector. Multiple factors related to housing and rearing may influence calf health in Danish dairy herds. In conventional herds calves are primarily housed in single pens the first 3–6 weeks after birth and then group housed. In organic herds calves are often pair-housed and then moved to group pens between 6 and 8 weeks of age. In both systems it in most common to feed colostrum manually within the first hours after birth. Milk feeding of calves usually consists of two daily feedings until weaning at 10–12 weeks with starter feed and hay on the side from the first week of life. While calf diarrhoea (CD) is the leading cause of mortality, bovine respiratory disease (BRD) causes the largest antimicrobial use in preweaned calves [1–3]. Risk factors for both disease complexes are numerous and range from low birth weight and failure of passive transfer (FPT) of colostral immunoglobulins as the first potential risk factors, to housing effects including group housing, ventilation, season and hygiene measures later in the calf life [4–6]. Since most of these risk factors seem to be controllable, improving calf health and mortality should be feasible. Further improvements rely on applicable monitoring tools that encompass both central risk factors and health measures. Previous monitoring tools have focused on a limited set of measures for selected outcomes such as clinical signs of respiratory or enteric disease, immunity, weight gain or mortality for the purpose of timely interventions such as recognition of clinical illness and treatment [7] or quality assurance [8]. One of the challenges of monitoring tools is that statistically grounded methods to detect changes in outcomes such as disease, weight gain and other production results are often not applicable in practice. Often, herds are operating with sample sizes too small to base any scientifically valid inference upon, as the number of calves in certain age groups can be very low due to production settings, e.g. selling of bull calves or moving heifers to rearing facilities. Additionally, variability between and within herds further contributes to the uncertainty of associations between given outcomes and risk factors or predictors when assessed locally in a given herd. Despite its challenges for outcome prediction, this variability is the key factor for improving herd health [9]. A variety of disease and performance monitoring tools have been developed for livestock based on statistical process control, an analytical approach utilizing routinely collected data on farm to enhance management processes and improve e.g. reproductive or udder health performance [10, 11]. To our knowledge, no previous attempts have been made to develop an integrative monitoring tool based on local evidence for calf health.

The objective of the present longitudinal study was to develop a feasible calf health-monitoring tool for use in dairy calf rearing practices from the first week of life until post weaning at the age of 12 weeks. This monitoring encompassed three key elements of calf health and robustness: Immunisation, health status and growth. Hence, initial immunisation status measured by serum Brix% and the subsequent health and recovery of sick calves was assessed by repeated clinical scoring together with weight gain estimated from measured heart girth circumference. Furthermore, pathogen detection was included as a potential add-on to the monitoring tool. The presented monitoring tool aims at easy visualization of different sources of information that describe calf robustness over time. Hence, the output is presented in a graphical manner to aid farmers in gaining a quick overview over the present state of their calves and to highlight potential patterns requiring attention and possible interventions.

## Methods

### Herd and calf selection

The nine study herds were selected based on convenience sampling according to their location and herd size aiming to be able to include enough calves in the study within the available resources. Three of the herds were located on Zealand, while the other six herds were located in the southern parts of Jutland, Denmark. In these nine herds, all heifer calves born within a three-week period in October 2018 were included. Calves were assessed at three time points: within the first ten days of life (1st week/T0), at 14–28 days (3rd week/T1) and finally at three months of age at day 90–110 (12th week/T2). These time points were chosen to reflect the most critical phases in the young calf’s life: the initial post-natal period (T0), where calves are highly susceptible to infections; the initial period with high risk of showing clinical disease (T1) upon completion of incubation periods for the most common pathogens; and finally, the high-risk period after weaning (T2).

### Blood sampling, body condition scoring, heart girth measurements and clinical scoring

Two trained observers performed assessments and scorings after having completed two training events. Each observer was assigned to a specific set of herds based on geography. At each herd visit 1–2 assistants, aiding in identifying, catching and restraining calves, handling samples (e.g. handling and labelling blood or faecal sample tubes) and entering registrations, accompanied the observers.

Blood samples were collected from the jugular vein, centrifuged, serum collected and frozen before sending them for further analysis at the research laboratory at Aarhus University (Tjele, Denmark). The serum IgG levels were assessed by use of refractometry. Serum IgG was categorized as either sufficient at Brix score equal to or above 8.1% or as failure of passive transfer at Brix scores below 8.1% [12].

All calves were assessed individually and housing type (i.e. single, pairwise or group housing) was assessed at every time point together with seven clinical measures. Body condition (BC) was scored by visual inspection at three levels (0: normal, 1: thin, 2: obese) [13] and weight of the calves was obtained by heart girth measurements using a measuring tape in cm and later converted to kilograms according to Heinrichs et al. [14]. Clinical observations included nasal and ocular discharge (0: none, 1: serous discharge, 2: mucopurulent or purulent discharge or crusts), coughing (0: no cough, 1: single cough manually provoked, 2: spontaneous coughing or repeated provoked coughs), faeces score (0: normal/pasty, 1: liquid or moderate amount of mucous, 2: profuse, watery or bloody stool or heavy amount of mucous), and hair loss on the rear, tail and/or hind legs (0: none, 1: present). Rectal temperature was registered and scored as 0: normal at values < 39 °C, 1: sub-febrile at 39–39.3 °C and as 2: febrile at ≥ 39.4 °C.

We constructed a respiratory disease (RD) variable from scores of nasal discharge, ocular discharge and cough based on the following classification: *Healthy (Score 0)*: Calves with no nasal discharge and no or serous ocular discharge and no or one induced cough. Additionally, calves with serous nasal discharge and no ocular discharge and no or one induced cough. *Severe (Score 2)*: Calves with simultaneous mucopurulent nasal and ocular discharge. Additionally, calves with repeated spontaneous coughs. *Mild (Score 1)*: All remaining calves.

We constructed a gastrointestinal disease (GD) variable from fecal scores and scores of hair loss [15] based on the following classification: *Healthy (Score 0)*: Calves with faecal score 0 and no hair loss at the subsequent observation time. *Severe (Score 2)*: Calves with faecal score 2. Additionally calves with faecal score 1 and hair loss at the subsequent observation time. *Mild (Score 1)*: All remaining calves.

Furthermore, we combined RD, GD and rectal temperature into a health status variable describing the primary condition of the calf. We used this in the visualization panel. Classification were as follows: *Healthy (H)*: Calves with no or mild respiratory disease and no or mild gastrointestinal disease and rectal temperature below 39.4 °C. *Gastrointestinal disease (G)*: Calves with no or mild respiratory disease and severe gastrointestinal disease. Additionally, calves with mild gastrointestinal disease and rectal temperature equal to or above 39.4 °C. *Respiratory disease (R)*: Calves with severe respiratory disease and no or mild gastrointestinal disease. Additionally, calves with mild respiratory disease and rectal temperature equal to or above 39.4 °C. *Both gastrointestinal and respiratory disease (B)*: Calves with severe respiratory disease and severe gastrointestinal disease. Additionally, calves with mild respiratory disease and mild gastrointestinal disease along with rectal temperature equal to or above 39.4 °C.

### Treatment data

Calf treatment data gathered from both treatments initiated by farmers or veterinarians depending during the study period were available for five of the nine herds and extracted from the Danish Cattle Database (SEGES Innovation P/S, Aarhus N, Denmark).

Only registrations of both antimicrobial and/or non-steroidal treatment related to diarrhoea (i.e. treatment of enteritis, diarrhoea, coccidiosis and cryptosporidiosis) and respiratory disease (treatment of pneumonia) were included.

### Diagnostic sample collection

All calves were subjected to a nasal swab and faecal sampling for pathogen detection by PCR at T0, T1 and T2 and blood sampling for IgG measurement at T0. Samples for pathogen diagnostics were handled as described previously [16, 17]. Pathogens assessed included influenza virus D, bovine corona virus, bovine respiratory syncytial virus (BRSV), *Mycoplasma* subspecies (spp), *Mycoplasma bovis*, *Histophilus somni*, *Mannheimia haemolytica*, *Pasteurella multocida* and *Trueperella pyogenes* in nasal swabs while faecal samples were analysed for bovine corona virus, rotavirus A, *Escherichia coli* F5*, Crysptosporidium, Eimeria bovis* and *Eimeria* subspecies. Samples were assessed as either positive based on a Cq value ≤ 25) or negative with Cq values > 25.

### Data management and summary

All data management and editing were performed in R version 4.0.1 [18]. The collected data were assessed descriptively by frequency statistics for the categorical variables regarding clinical scores, while the continuous variables such as rectal temperature, calculated body weight in kg, serum Brix% and number of animals in pen were summarised by mean and standard deviation (SD given as ±).

### Visualisation tool for health monitoring within herds

**Fig. 1:**
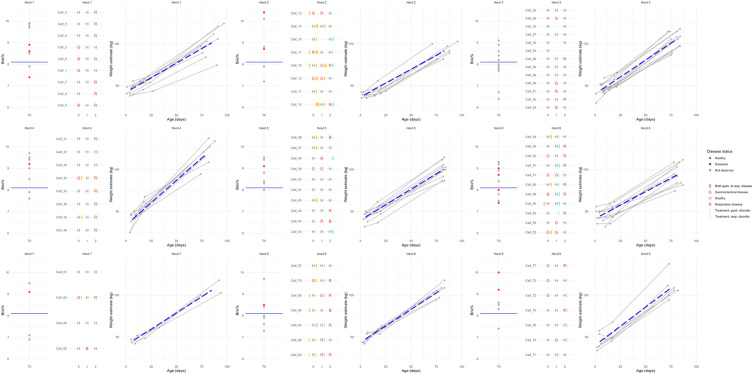
Health monitoring of 77 heifer calves from nine Danish dairy herds. The first panel shows the immunisation status (Brix%) classified as sufficient (Brix% ≥ 8.1) or as failure of passive transfer (Brix% < 8.1) combined with the calf´s initial heath status assessed in the first week of life (T0). The second panel summarises the health status based on clinical scoring (*H* Healthy, *G* Gastrointestinal disease, *R* Respiratory disease, *B* Both gastrointestinal and respiratory disease and treatments) and treatments (yellow = gastrointestinal disorder; blue = respiratory disorders) within the first 12 weeks of age. The third panel depicts the estimated growth compared to herd mean (blue dashed line)

We constructed a dashboard (Fig. 1) for each herd consisting of three panels. The first panel visualised immunity and health status of the calves at T0. We plotted each calf as a point on a vertical axis according to Brix%. Diseased calves were highlighted in red and the FPT threshold was displayed as a horizontal blue line. The second panel visualised the health status of the calves for the three critical time points (T0, T1 and T2) and treatments in the intermediate periods. Calves observed with either RD, GD or both, as classified from the clinical scores, were highlighted in red. Calves were ranked by immunity status (Brix%) to facilitate comparison to the first panel. Treatments were displayed as vertical lines and coloured yellow if related to diarrhoea and light blue if related respiratory disease. The third and final panel visualised weight gain through a scatterplot of weight estimates against age of the calf. For each calf, lines connected the weight estimates and the herd average was displayed as a blue dashed line.

**Fig. 2:**
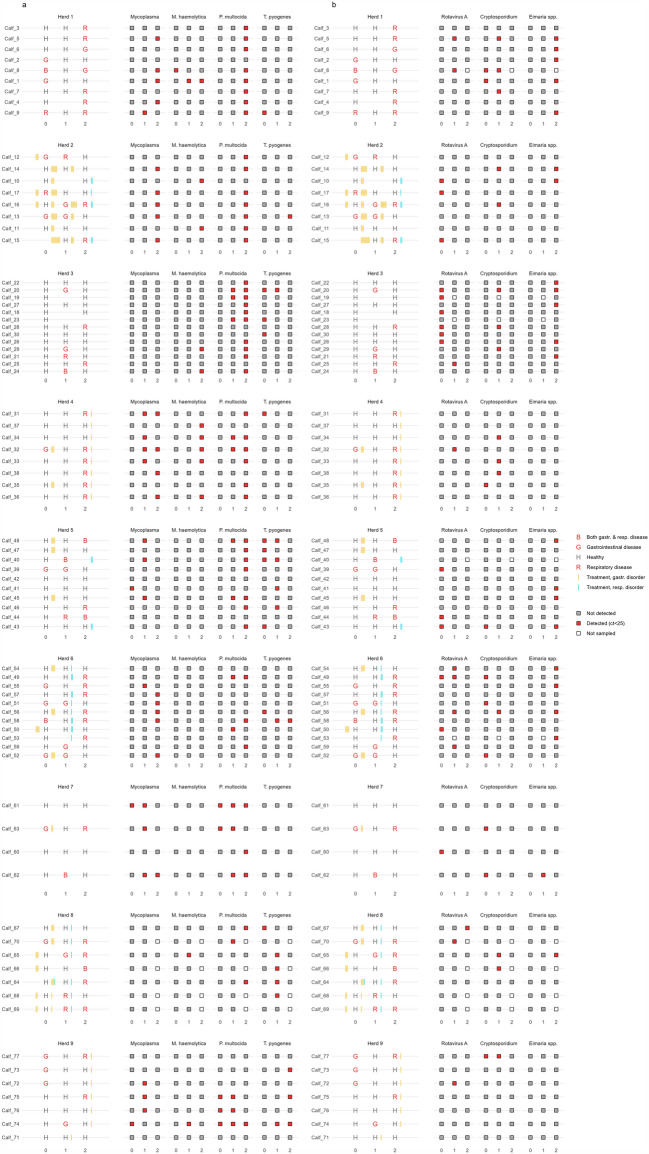
Visualisation of disease and pathogens detected over time. The left panel shows the single calf´s health status (*H* Healthy, *G* Gastrointestinal disease, *R* Respiratory disease, *B* Both gastrointestinal and respiratory disease and treatments) and treatments (yellow = gastrointestinal disorder; blue = respiratory disorders). The panel on the right gives an overview of the rt-PCR test results found in a) nasal swabs and b) faecal samples. Red boxes represent positive tests for the given pathogen at Cq-values ≤ 25. Grey boxes represent negative test results (Cq > 25)

For visualising pathogen detection, we constructed a secondary dashboard with two panels. The first panel was identical to the central panel in the primary dashboard visualising health status and treatment of the calves. The second panel displayed for each pathogen, whether the calf was positive (red) or negative (grey) for the respective pathogen at each critical time point. A similar dashboard was constructed for both respiratory (Fig. 2a) and enteric pathogens (Fig. 2b).

## Results

Descriptive results from the key components in the monitoring of calf health and growth are presented in the following section followed by an evaluation of the central output given by the monitoring of single calves within the nine herds presented in Fig. 1.

### Population

A total of 82 heifer calves were born between September and October 2018 and enrolled in the longitudinal sampling in the nine included dairy herds. Out of the initial 82 heifer calves, 77 calves had complete registrations for all three assessments. Two calves died and one farm sold three heifers within the study period. Study demographics are shown in Table 1. Herd size ranged from 47 to 460 cows at a mean herd size of 178 cows. The overall distribution of breeds was 84.4% (n = 65) Danish Holstein, 9% (n = 7) dairy cross breeds and 6.6% Danish Red (n = 5). All nine herds were conventional dairy herds with a mandatory herd health program by the herd veterinarian. Mean age of calves at T0 was 5 days (min 1; max 11), 19 days (min 14; max 33) at T1 and 81 days (min 71; max 97) at T2. All calves assessed at T0 were housed in individual pens, while 86% of the calves were still housed individually by T1 and the remaining 14% had been moved into group pens. By T2 all calves except six calves in herd 9 were housed in group pens, whilst these six were still housed individually. Herds providing group housing for calves at T2 kept an average of 8.3 ± 4.2 calves per pen. Calves were fed milk until gradual weaning commenced around 10th to 12th week of life supplemented by calf muesli and fresh water.

**Table 1:** Herd descriptive statistics for nine Danish dairy herds enrolled in the calf health monitoring study

Herd	Time	N calves	Brix %	N FPT^a^	N Diseased	Weight (kg)	Estimated weight 56d (kg)	Estimated ADWG^b^ T0—56d (g)
1	T0	9	8.81 ± 0.86	2 (22%)	4 (44.4%)	45.3 ± 6.0		
	T1	8			0 (0.0%)	53.6 ± 5.8		
	T2	9			7 (77.8%)	101.1 ± 16.6	79.8 ± 10.2	670 ± 137
2	T0	7	8.49 ± 1.27	2 (25%)	3 (42.9%)	39.0 ± 2.7		
	T1	7			3 (42.9%)	44.3 ± 5.6		
	T2	8			2 (25.0%)	91.3 ± 8.1	71.9 ± 7.0	630 ± 101
3	T0	13	7.89 ± 0.81	6 (46%)	0 (0.0%)	45.5 ± 7.2		
	T1	10			4 (40.0%)	54.0 ± 6.6		
	T2	12			2 (16.7%)	104.0 ± 10.6	84.6 ± 8.7	790 ± 103
4	T0	8	8.85 ± 0.77	2 (25%)	1 (12.5%)	40.3 ± 10.0		
	T1	8			0 (0.0%)	56.8 ± 6.9		
	T2	8			6 (75.0%)	118.6 ± 14.2	94.4 ± 6.7	750 ± 214
5	T0	10	8.78 ± 0.59	1 (10%)	1 (10.0%)	43.3 ± 5.5		
	T1	10			3 (30.0%)	51.8 ± 6.9		
	T2	9			3 (33.3%)	100.0 ± 9.6	80.3 ± 7.7	730 ± 85
6	T0	11	8.38 ± 0.68	4 (36%)	4 (36.4%)	44.4 ± 7.2		
	T1	10			3 (30.0%)	51.3 ± 8.6		
	T2	11			6 (54.5%)	94.0 ± 12.5	74.8 ± 10.5	590 ± 136
7	T0	4	8.15 ± 1.34	2 (50%)	1 (25.0%)	46.1 ± 4.3		
	T1	4			1 (25.0%)	57.7 ± 6.6		
	T2	4			1 (25.0%)	105.9 ± 8.0	82.2 ± 3.7	750 ± 155
8	T0	7	8.2 ± 0.78	4 (57%)	1 (14.3%)	47.7 ± 5.2		
	T1	7			3 (42.9%)	59.5 ± 3.6		
	T2	7			5 (71.4%)	105.0 ± 7.8	87.7 ± 3.7	780 ± 91
9	T0	7	8.86 ± 0.94	1 (14%)	3 (42.9%)	44.5 ± 8.6		
	T1	7			1 (14.3%)	52.3 ± 7.8		
	T2	7			2 (28.6%)	109.1 ± 13.7	90.3 ± 12.3	890 ± 192

^a^ *FPT* Failure of passive transfer, ^b^ *ADWG* Average daily weight gain

### Immunisation

The distribution of serum Brix% showed a mean of 8.5% ± 0.9%. Although, mean serum Brix% was indicative of sufficient immunisation within-herd Brix% levels showed large variations with within-herd means ranging from as low as 6.4% to a maximum of 10%. Within-herd prevalence of FPT ranged from 10 to 50%, with 24 out of the 77 (31%) calves having FPT.

### Clinical scores

The distributions of clinical scores and disease variables are given in Table 2. At T0, most frequent signs of disease were presented by mild RD 46% with nasal discharge score 1 being the most prominent clinical sign at a prevalence of 51%, while 18% showed more severe signs of nasal discharge (score 2), but no spontaneous cough was present. Profuse diarrhoea presented by faecal score 2 was seen in 13% of the calves at T0. Additionally, five calves with faecal score 1 at T0 had hair loss at T1 and thus 20% of calves were classified with severe signs of GD at T0. At T1 mild signs of RD were found in 56% of the calves with 44% nasal discharge at score 1, while 4% of the calves showed more severe signs of RD with 16% nasal discharge score 2 and 4% spontaneous cough. Profuse diarrhoea was seen in 8% of the calves at T1 and including milder cases with subsequent hair loss the prevalence of severe GD (11.7%) at T1 was lower than observed at T0.

**Table 2:** Frequencies of clinical scores and disease variables

Clinical variable	Time point	Score 0 (%)	Score 1 (%)	Score 2 (%)	NA (%)
Body condition	T0	68 (88.3)	9 (11.7)		
	T1	66 (85.7)	11 (14.3)		
	T2	69 (89.6)	8 (10.4)		
Nasal discharge	T0	24 (31.2)	39 (50.6)	14 (18.2)	
	T1	31 (40.3)	34 (44.2)	12 (15.6)	
	T2	6 (7.8)	21 (27.3)	50 (64.9)	
Ocular discharge	T0	56 (72.7)	18 (23.4)	3 (3.9)	
	T1	46 (59.7)	14 (18.2)	17 (22.1)	
	T2	20 (26)	24 (31.2)	33 (42.9)	
Cough	T0	76 (98.7)	1 (1.3)		
	T1	72 (93.5)	2 (2.6)	3 (3.9)	
	T2	62 (80.5)		15 (19.5)	
Faeces score	T0	42 (54.5)	24 (31.2)	10 (13)	1 (1.3)
	T1	23 (29.9)	42 (54.5)	6 (7.8)	6 (7.8)
	T2	43 (55.8)	27 (35.1)	5 (6.5)	2 (2.6)
Hair loss	T0	77 (100)			
	T1	71 (92.2)	6 (7.8)		
	T2	72 (93.5)	5 (6.5)		
Temp	T0	50 (64.9)	20 (26)	7 (9.1)	
	T1	53 (68.8)	15 (19.5)	9 (11.7)	
	T2	49 (63.6)	19 (24.7)	9 (11.7)	
GD	T0	41 (53.2)	20 (26)	15 (19.5)	1 (1.3)
	T1	23 (29.9)	39 (50.6)	9 (11.7)	6 (7.8)
	T2	43 (55.8)	27 (35.1)	5 (6.5)	2 (2.6)
RD	T0	40 (51.9)	35 (45.5)	2 (2.6)	
	T1	31 (40.3)	43 (55.8)	3 (3.9)	
	T2	4 (5.2)	41 (53.2)	32 (41.6)	

The prevalence of respiratory disorders increased in calves after grouping at T2, where signs of mild RD was found in 53% and severe RD in 42% of the calves. Severe GD was further reduced at T2 (7%). Prevalence of febrile calves as well as lean calves tended to be consistent across age groups. Ocular discharge followed a similar pattern as nasal discharge with increasing prevalence at increasing age: T0 23% mild and 4% severe cases; T1 18% mild and 22% severe cases; T2 31% mild and 43% severe cases, respectively.

### Growth

The included herds had an ADWG of 780 g ± 174 g from T0 to T2, which would result at an average estimated weight week 8 (age 56 days) of 84.5 kg ± 10.5 kg meaning an ADWG of 760 g ± 190 g over the calves first eight weeks of life with variations between herds.

### Health monitoring within herds

Figure 1 illustrates the visualization tool for the nine herds revealing large variation between herds across all parameters included. Within-herd prevalence of diseased calves (R, G and both) at T0 varied from 0% in herd 3 to 44% in herd 1 at T0. However, the prevalence of calves with FPT is inverse with 6/13 with FPT in herd 3 compared to only 2/9 in herd 1. Despite the higher proportion of calves with FPT at T0, herd 3 achieved lower numbers of sick calves and not only had a more centred but higher estimated ADWG throughout to T2 indicating good calf management. In contrast, although calves recovered from their initial disease at T0 and no calves were scored ill at T1 in herd 1, the disease prevalence increased dramatically after regrouping and weaning calves at T2, indicating challenges in the management of these stressful events. However, all herds had the highest prevalence of diseased calves at T2 (range 17–78%) compared to T0 and T1. Treatment data on calf level were not available for herds 1 and 3.

Finally, weight estimates plotted in Fig. 1 show large variation in weight gain and birth weight within-herds, with some herds showing low within-herd variation in ADWG (herd 7 and 8) compared to others (e.g. herd 1).

### Pathogen profiles

Results from the pathogen detection showed substantial variation in the presence of pathogens across herds (Fig. 2a, b). Most prominent pathogen detected from nasal swabs in young calves at T0 and T1 was *T. pyogenes* while *P. multocida* and *Mycoplasma* spp primarily were detected amongst older calves (T2). Faecal pathogens found at T0 and T1 were dominated by presence of rotavirus A and *Cryptosporidium* with a shift towards *E. bovis* and *Eimeria* spp with increasing age (Table 3).

**Table 3:** Pathogens detected in nasal swab samples from 77 heifer calves in nine Danish dairy herds

Pathogen	Time point	Positive samples (%)	N positive herds
Bovine corona virus	T0		0
	T1		0
	T2	2 (2.6)	2
BRSV	T0		0
	T1		0
	T2		0
*Histophilus somni*	T0		0
	T1	1 (1.2)	1
	T2	4 (5.2)	3
Influenza virus D	T0		0
	T1		0
	T2	2 (2.6)	1
*Mycoplasma bovis*	T0		0
	T1	1 (1.2)	1
	T2	2 (2.6)	1
*Mannheimia haemolytica*	T0	1 (1.2)	1
	T1	4 (4.9)	2
	T2	10 (13)	4
*Mycoplasma* spp.	T0	3 (3.7)	3
	T1	15 (18.3)	6
	T2	16 (20.8)	5
*Pasteurella multocida*	T0	5 (6.1)	2
	T1	16 (19.5)	6
	T2	47 (61)	9
*Truepurella pyogenes*	T0	11 (13.4)	6
	T1	11 (13.4)	6
	T2	5 (6.5)	3

## Discussion

Calf health monitoring is laborious and tedious work, but when done systematically and regularly, it can provide evidence for e.g. detecting shortcomings in the rearing process, for evaluating interventions (e.g. treatment), for quality assurance and decision-making. The present study introduced a basic protocol covering feasible and practical measures for calf health based on clinical scoring, immunisation, weight measurements and pathogen detection. By combining real-time data with historical data in one visualisation panel and adding information on diagnostics possible patterns of potential health hazards in a herd can be detected more timely. In addition, the choice of treatment could be based on local evidence, favouring the general policies on reducing anti-microbial resistance and enhancing animal welfare. Our approach showed substantial variance in between-herd disease prevalence among dairy heifer calves as shown in Table 2, emphasizing the need for within-herd comparisons as presented in Fig. 1. Inclusion of available treatment registrations allows for an assessment of the herd specific treatment strategy. Combined with clinical observations, we may relate the treatment intensity to the overall health status of the calves. This would allow for the evaluation whether good calf health is mainly related to management or intensive treatment, or whether poor calf health persists despite intensive treatment. The presented visualisation of each herd is based on repeated observations of calves over time, which easily detects individuals presenting exceptional variation within the herd, and which might need additional diagnostic procedures or targeted intervention. The real-time aspect of the monitoring might also allow for a better understanding of potential causes for occurrence of outliers within a herd. The approach could be further adjusted by implementing herd-specific warning and alarming thresholds to e.g. IgG levels or disease or pathogen prevalence. In order to monitor immunisation and pathogen patterns in the herd, calves need to be restrained and blood samples need to be taken by authorized personnel (e.g. a veterinarian), making this part of the health monitoring less practically feasible. A similar approach monitoring pathogen profiles within herds over time is proposed for nursery and finisher pigs [19]. However, the clinical screening and weight estimations based on tape measuring are very useful and practical tools, which easily could be implemented in the daily routines and add value to the calf rearing process.

Immunisation was not sufficient in 31% of the 77 calves, leaving 69% with sufficient immunisation, which is very close to the reported success rate of 73.3% in Holstein heifers reported by Shivley et al*.* [20]. More than half of the study herds showed a large variation of serum IgG levels, which could be improved by focusing on the quality of colostrum fed and the time of first feeding. Nonetheless, all herds showed good mean levels but when compared with the longitudinal aspect visualised in the herd panels, a possible correlation between FPT rate and disease rate could be suspected and further confirmed by inclusion of more calves.

Presented prevalence results are in accordance to previous longitudinal studies [4, 5, 20, 21], as gastrointestinal diseases are described as the primary cause for morbidity in the first weeks of life. In the present study 20% of the enrolled calves showed severe signs of GD and 2.6% RD at T0. While GD prevalence declined over time (T1: 12%, T2: 7%), prevalence of severe RD increased with age from 4% at T1 to 42% at T2. Only four of the 77 calves were classified with GD at both T0 and T1, indicating that only few calves suffer from prolonged periods with gastrointestinal disease. Mahendran et al*.* [21] who compared weekly pre-weaning health scores with mortality and production parameters also found similar disease prevalence within the first eight weeks of the dairy calves’ life, where the most frequent combinations of clinical signs were pyrexia/ocular discharge (6.96%) and pyrexia/coughing (1.9%), whereas pyrexia/ diarrhoea was less frequent (1.1%). Additionally, they found a significant association between FPT and pyrexia, which the present study could not confirm maybe due to the higher threshold for FPT at 8.1 g/dL compared to Mahendran et al. [21] threshold of 5.2 g/dL by refractometer (Brix%). Since mortality was higher in calves that had not shown pyrexia throughout the study period, they concluded that weekly assessments were not sufficient for detecting acute diseases with high mortality. This issue about the timeliness also plays a role in the present study. Danish herds are increasing in size, but not necessarily proportionally in the number of employees, leaving little time for such systematic assessments of the calves. The time lapse between T1 and T2 and the limit of three time points of observations per calf in the present study limit the possibilities to assess association, since we cannot account for eventual disease episode between the time points here. While McGuirk et al. [7] suggest screenings twice weekly for the early detection of respiratory disorders; a selection of calves for examination must be made for farm workers to be able to meet the screening intervals on top of the daily duties. The present study was part of a larger inter-institutional research project. Hence, the study design had to accommodate the given financial constraints. Based on our findings, weekly visits would have been the best monitoring interval. Especially for meeting the purpose of cost-effective monitoring, a more comprehensive data collection should be evaluated in future studies. Nonetheless, since herd routines vary a lot, stating monitoring intervals might discourage farmers to use the monitoring tool, as they might not implement a too ambitious monitoring scheme for their particular herd. However, by leaving the choice of interval up to the farmers, they decide what level of data quality is produced. The herd veterinarian could be included in this discussion to ensure the best possible level of data quality. Hence, the present study enables farmers to choose a given examination interval, where more frequent assessments within the pre-weaning period until a couple of weeks post-weaning might be advisable, based on the present findings and on the purpose of the screening e.g. evaluation of interventions or treatment. Another limitation of the present study was the relatively small sample of the included nine cohorts of heifer calves. In order to validate the present monitoring tool, more cohorts from the same herds should be monitored to provide a sort of baseline pattern for the individual herd over time and across seasons. All cohorts were followed during the same period, but seasonality might have an influence on morbidity when this is assessed continuously.

The presented pathogen distributions are in alignment with previous studies showing bovine rota virus and *Cryptosporidium* spp. as the major agents for neonatal diarrhoea [21, 22, 24], while coccidiosis was the major finding in post weaned calves. In the present study only 20/45 calves with severe signs of GD also had a concurrent positive test for enteric pathogens. This highlights the importance of the multi-factorial causes of GD such as colostrum management, hygiene and milk feeding strategies also mentioned by Meganck et al. [23]. In regards to RD, *Mycoplasma* infections were the main infectious causes accompanied by *P. multocida* and *T. pyogenes* as the main bacterial agents. While BRSV and bovine parainfluenzavirus type-3 dominated the viral component of respiratory disorders in Danish dairy calves in previous decades [25, 26], the recently increased use of vaccines might have proven efficient and shifted the pathogen pattern. The secondary bacterial infections also presented themselves with severe signs of RD and an expected increase in clinical cases also testing positive for pathogens at T2. These findings might imply that a more targeted approach towards antimicrobial treatment can be performed by including pathogen screening, as the younger age groups receiving antimicrobial treatment in the present study might not have benefitted from antimicrobial use and could have been treated with NSAID´s for the potential initial viral infection instead [27–29]. Nonetheless, pathogen detection is highly depended on the diagnostic properties of the laboratory tests applied.

## Conclusions

The large variation in both clinical disease and pathogen occurrence across herds emphasizes the need for herd specific interventions. Combining the results of the present study from measures of immunisation, health and growth from individual calves in one visualisation panel allowed for the detection of patterns across age groups in the specific herds. This could potentially enable calf caretakers to detect emerging infections and initiate interventions more timely as well as to evaluate the effect of given interventions. Due to the focus on practicality and feasibility, the tool could also provide quality assurance of calf rearing and management practices. Although validation of the presented monitoring tool is still needed, the proposed systematic approach could still contribute to enhance and ensure better calf health and welfare as it provides an intuitive graphical overview of the most important calf rearing processes in the given herd.

## Data Availability

The datasets used and analyzed during the current study are available from the corresponding author on reasonable request.

## Declarations

### Prior publication

Data have not been published previously.
